# Partial Thrombosis of Inferior Mesenteric Vein With Thrombophlebitis

**DOI:** 10.7759/cureus.16900

**Published:** 2021-08-05

**Authors:** Richard Medina-Perez, Daniel J Campbell, Jose Mario Acosta Rullan, Sheyla Gonzalez

**Affiliations:** 1 Internal Medicine, Aventura Hospital and Medical Center, Aventura, USA; 2 Internal Medicine, Aventura Hospital and Medical Center, Miami, USA

**Keywords:** venous thrombus, rare, mesenteric vein, inferior mesenteric, ct angiography, delayed phase, thrombophilia, capture delayed images, thrombophlebitis

## Abstract

Inferior mesenteric vein thrombosis (IMVT) is a rare entity that can lead to a potentially lethal event unless recognized early in the disease. Although its prevalence is low, IMVT presents mainly in certain conditions such as in inflammatory processes like diverticulitis, arrhythmias, hypercoagulable states, connective tissue disorders, malignancy, or hereditary thrombophilias.

Mesenteric venous thrombophlebitis is a condition in which a blood clot in a vein causes inflammation and pain. It can appear in an acute or subacute manner that leads to acute mesenteric ischemia. The case of a 58-year-old male without a significant past medical history who presented with suprapubic abdominal pain secondary to a partial IMVT of unknown etiology with accompanying thrombophlebitis is discussed here.

## Introduction

Acute mesenteric vein thrombosis (MVT) is a rare but an important cause of intestinal ischemia, and an early diagnosis requires a high index of suspicion. Diagnosing acute mesenteric ischemia (AMI) is challenging, and failure to recognize it prior to the onset of intestinal necrosis is associated with high mortality. AMI can be caused by various conditions such as arterial occlusion, venous occlusion, strangulating obstruction, and hypoperfusion associated with non-occlusive vascular disease.

Early diagnosis and prompt treatment are goals of successful therapy but there are not any randomized control trials to guide treatment [1]. The reported incidence of MVT is 2.7 per 100,000. The superior mesenteric vein (SMV) is involved in approximately 95% of all cases, with only 5% of reported MVTs being found in the inferior mesenteric vein (IMV) for reasons that are not well understood. Although IMV thrombosis (IMVT) has a very low prevalence, its reported mortality ranges from 15% to 23%. Immediate anticoagulation is warranted given the significant morbidity and high mortality risk [2]. 

Although there are not any specific clinical features of AMI, a high index of suspicion is necessary for the evaluation and diagnosis of MVT. Definitive diagnosis is made by imaging studies that reveal the presence of thrombosis within the mesenteric veins. It is recommended that the use of computed tomography (CT) with and without intravenous (IV) or oral contrast is used for initial screening of MVT. More reliable modalities like CT angiography (CTA) and magnetic resonance venography are also helpful; however, the ability of CT for diagnosing mesenteric ischemia has recently been reported to have a sensitivity of approximately 90%. The CTA has the added benefit of capturing delayed images in the venous phase, especially in the setting of non-diagnostic non-contrast CT, which leads to a high clinical suspicion of MVT [3].

## Case presentation

A 58-year-old male with no significant past medical history presented to our institution for progressively worsening suprapubic pain lasting one week and not associated with urination. One week earlier he had presented with similar symptoms and complaints including general myalgias, dark urine, and subjective fevers. He was sent home with a course of antibiotics without much improvement for presumptive urinary tract infection.

He returned on this admission with persistent suprapubic pain described as sharp, non-radiating with a 10/10 severity. Pain was exacerbated by activity and was alleviated with rest and the use of non-steroidal anti-inflammatory drugs. He denied fevers, chills, night sweats, nausea, vomiting, or changes in bowel habits. At presentation the patient was hemodynamically stable. Physical examination was significant for abdominal tenderness out of proportion with the presence of voluntary guarding. The abdomen was soft, non-distended with normoactive bowel sounds. The rest of the examination was unremarkable.

**Figure 1:**
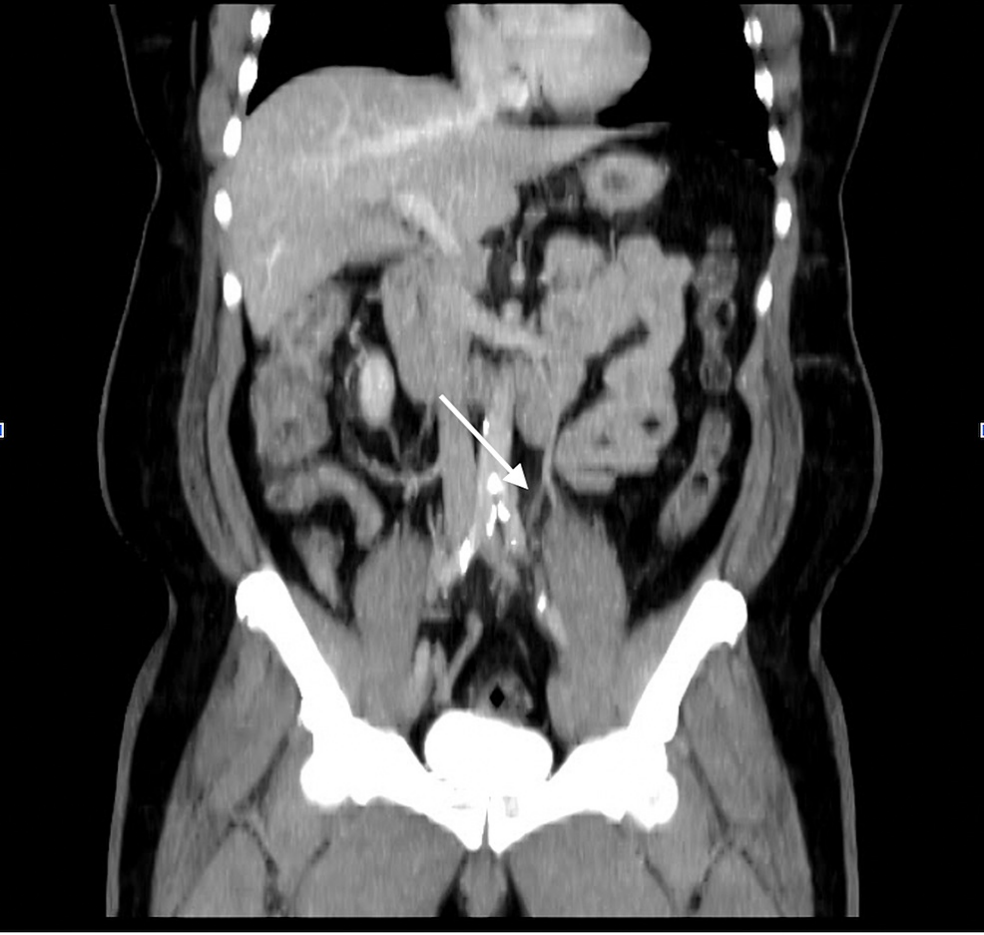
Reformatted coronal view CT abdomen and pelvis with delayed phase showing (arrow) inflammatory changes surrounding the area of partially occluded inferior mesenteric vein. CT, computed tomography.

**Figure 2:**
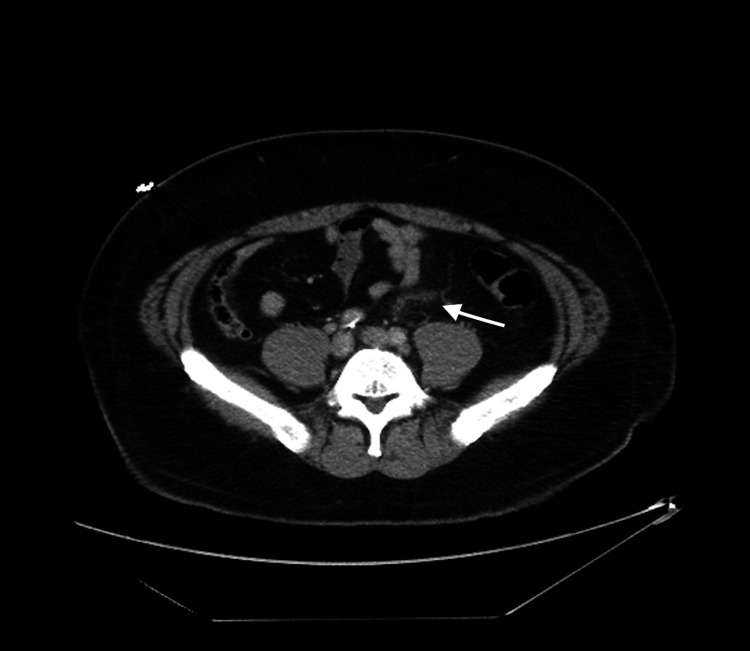
Transverse view CT abdomen and pelvis with delayed imaging to capture venous phase showing (arrow) inflammation surrounding the inferior mesenteric vein concerning for thrombophlebitis. CT, computed tomography.

Initial blood count revealed a pertinent result of hemoglobin 15.5 grams/deciliter and leukocyte count of 13.2 cells/liter. A comprehensive chemistry panel revealed an elevated alanine aminotransferase of 66 units/liter but with otherwise unremarkable results including lactic acid of 0.9 millimoles/liter and severe acute respiratory syndrome coronavirus 2 polymerase chain reaction negative. The urinalysis was significant for urine specific gravity of 1.056. Imaging studies included a CT IV contrast of the abdomen and pelvis, which was significant for prominence of the IMV, with surrounding mesenteric inflammation, raising concerns for IMVT versus thrombophlebitis. As a result of these imaging findings, general and vascular surgery were consulted and recommendations to place the patient on full-dose anticoagulation with lovenox were given, in addition to obtaining a CTA with delayed imaging in order to capture the venous phase of the IMV. The CTA confirmed a partial occlusion of the IMV with surrounding inflammatory changes to both the portal vein and SMVs, which were patent (Figures 1, 2).

Given the additional concern for the unusual location of the thrombus, a hypercoagulable workup was ordered. Hematology oncology was consulted as well to determine if there was a hereditary predisposition to clotting. After a few days of treatment with anticoagulation and supportive care, his symptoms improved and surgical intervention was no longer indicated. The patient was safely discharged on oral anticoagulation with eliquis and advised to follow up in the clinic for further workup. On further investigation in the outpatient clinic, hypercoagulable workup was ordered including protein C and S deficiency, antithrombin III deficiency, Factor V Leiden mutation , plasminogen activator inhibitor, prothrombin 20210 mutation, and flow cytometry for paroxysmal nocturnal hemoglobinuria, which where all negative. Patient was advised to continue oral anticoagulation indefinitely due to unprovoked IMVT. 

## Discussion

The IMV is a continuation of the superior rectal vein. Its sole purpose functions to drain the distal transverse colon, descending colon, and rectum. Thrombosis of the IMV or any of the mesenteric veins can lead to decreased perfusion pressures that can result in significant bowel edema. This can often lead to the stagnation of blood flow and consequently cause submucosal hemorrhage or (in the case of complete venous return occlusion) bowel infarction. 

IMVT is a relatively uncommon finding that has been reported to be responsible for only 4%-11% of cases of AMI and carries a 15%-23% risk of mortality [3]. Clinical presentation can vary, and tends to be vague and non-specific. Symptomatic presentation can include (but is not limited to) abdominal pain, nausea, vomiting, hematemesis, melena, constipation, or diarrhea. If not promptly recognized, however, it can lead to severe complications such as peritonitis, intestinal infarction, or even death. 

MVT and AMI are both associated with prothrombotic states (i.e. atrial fibrillation, atherosclerosis, and other thrombophilias); however, the diagnosis has been made without these associated pathologies present [4]. It is estimated that the sensitivity for AMI diagnosed with CT imaging is approximately 90% [5]. Radiological signs of MVT include vascular changes such as filling defects, congestion, and stranding, which can extend to nearby vasculature. Signs of intestinal ischemia include mesenteric edema, small bowel edema, wall thickening, dilated bowel loops, intestinal pneumatosis, and ascites [6]. 

Treatment of symptomatic patients with radiological signs of acute MVT will be determined by a few factors such as 1) the presence or absence of clinical signs of peritonitis or 2) overt signs of intestinal necrosis or perforation on imaging. Any positive findings of the aforementioned will determine the need for a surgical intervention versus non-surgical intervention [7]. Conservative management varies on a case-by-case basis, but generally consists of measures such as bowel rest, pain control, IV hydration, anticoagulation, and serial abdominal examinations like in our presented case. Case reports have demonstrated that both management options yield similar results in regard to morbidity, mortality, and survival rates. When compared to surgical management, non-surgical management was associated with a shorter duration of hospital stay [8].

## Conclusions

Our case of a partial IMVT with associated thrombophlebitis is seldom reported in the literature. Relevant history, physical examination, and high clinical suspicion are crucial in these cases. When clinical suspicion of mesenteric thrombosis or thrombophlebitis is present, a CT scan with IV contrast is the recommended initial imaging study of choice. Early recognition of mesenteric thrombosis is essential as it can lead to AMI, which is associated with high morbidity and mortality rates. Given the vast array of clinical presentations, we believe further research is warranted to raise awareness of these pathologies. 
